# Nivolumab-Induced Diabetic Ketoacidosis: A Case Report

**DOI:** 10.7759/cureus.64319

**Published:** 2024-07-11

**Authors:** Drake Giese, Ali Elkhedr, Geethu Jnaneswaran, Katherine M Ernste

**Affiliations:** 1 Orthopedics, Medical College of Wisconsin, Milwaukee, USA; 2 Internal Medicine, Marshfield Medical Center, Marshfield, USA; 3 Family Medicine, Medical College of Wisconsin, Milwaukee, USA

**Keywords:** immunomodulatory therapy, monoclonal antibody, autoimmune disease, diabetic ketoacidosis, checkpoint inhibitor, nivolumab

## Abstract

Nivolumab, an investigational monoclonal antibody targeting a specific immune pathway, has shown promise in treating various autoimmune diseases. However, like other immunomodulatory agents, it has potential side effects. This case report describes a rare adverse event of nivolumab-induced diabetic ketoacidosis (DKA) in a patient with a history of adrenal insufficiency secondary to nivolumab. The patient presented with symptoms of hyperglycemia, metabolic acidosis, and ketosis after receiving nivolumab therapy for 12 cycles. Prompt recognition and management of nivolumab-induced DKA are crucial to prevent complications and ensure patient safety.

## Introduction

Immune checkpoint inhibitors (ICIs) have been monumental in advancing cancer therapy and are increasingly common treatments for various malignancies [1-4]. One subgroup of ICIs called programmed death-1 (PD-1) inhibitors is becoming the standard of care for various malignancies including malignant melanoma and non-small cell lung cancer [2-3].

PD-1 inhibitors, such as nivolumab, act by blocking signals between immune cells and cancer cells, which augments the body’s T-cell-mediated destruction of tumor cells [5]. As this mechanism involves direct interaction with the inherent immune system of the human body, there have been documented complications and side effects referred to as immune-related adverse events [6]. These adverse events depend on the drug's class and dose, in addition to the type of cancer and individual factors related to the patient [6]. Recent studies suggest that endocrine dysfunction following ICI therapy is approximately 12% and the development of diabetes mellitus is an extremely rare complication [7,8].

To minimize mortality and prevent complications there should be high clinical suspicion for adverse effects in patients receiving nivolumab. Prompt diagnosis and therapeutic interventions are essential for preventing life-threatening side effects. Here, we describe the occurrence of nivolumab-induced diabetic ketoacidosis (DKA) in a patient with a history of autoimmune dysfunction secondary to nivolumab.

## Case presentation

A 68-year-old male with coronary artery disease (CAD) status post coronary artery bypass (CABG), chronic kidney disease (CKD), melanoma with metastasis to the brain on chemotherapy and adrenal insufficiency secondary to nivolumab, was transferred from vascular surgery two days after an iliofemoral arterioplasty with delirium, adrenal crisis, and euglycemic ketoacidosis. On presentation on September 16, 2023, the patient was hypotensive and tachypneic. On examination before transfer from vascular surgery, the patient had dysarthria, diplopia, and weakness of extremities. Imaging including CT head, CTA head and neck, CT cerebral perfusion, and MRI was negative for any acute intracranial pathology including stroke. Labs revealed high anion gap metabolic acidosis, elevated blood glucose, elevated beta-hydroxybutyrate, and unremarkable lactic acid levels (Table 1). Other findings unrelated to his current issue included anemia likely related to his recent procedure (Table 1).

Based on the patient's clinical presentation and laboratory findings, a diagnosis of DKA was made. The temporal relationship between nivolumab initiation and the onset of DKA raised suspicion of drug-induced etiology. Other causes of DKA, such as infection or insulin deficiency, were ruled out. The patient received IV dexamethasone for primary adrenal insufficiency also considered secondary to nivolumab. The patient was started on intravenous insulin therapy, fluid resuscitation, and electrolyte replacement and his clinical condition gradually improved with intensive management. Soon after initiation of therapy, his beta-hydroxybutyrate began trending down, his blood glucose levels normalized, and his metabolic acidosis resolved (Table 1). He did have hypokalemia secondary to insulin therapy which resolved with IV replacement. Subsequently, the patient was transitioned to subcutaneous insulin therapy.

**Table 1:** Lab values during hospitalization

Labs	9/16/2023 15:37	9/17/2023 20:12	9/18/2023 07:34	9/19/2023 20:30
Potassium (mmol/L)	4.4		3.6	2.9
Bicarbonate (mmol/L)	10.0		16	24
Glucose (mg/dL)	210.0		139	129
Anion gap (mmol/L)	17.0		12	10
Beta-hydroxybutyrate (mmol/L)	8.2	0.38		
Lactic acid (mmol/L)	0.8			
Hemoglobin (g/dL)	10.5			
Hematocrit	31.4%			

During follow-up visits, the patient's diabetes management was optimized, and he remained stable with no recurrent episodes of DKA. Nivolumab was cautiously resumed due to the patient’s melanoma.

## Discussion

One rare, but well-documented side effect of PD-1 inhibitors, such as nivolumab, is endocrinopathies such as hypothyroidism, panhypophysitis, and central and secondary adrenal insufficiency [3,4,6]. A recent meta-analysis of 38 random clinical trials found that endocrine disorders following treatment with checkpoint inhibitors such as PD-1 inhibitors occur in just 12% of cases [7]. Many of these cases reported effects on the thyroid or pituitary gland [7].

An exceedingly rare side effect that is poorly represented in the literature is insulin-dependent diabetes, reported to be as low as 1-2% of patients receiving PD-1 therapy [9,10]. In a large case study, over 76% of cases of PD-1 inhibitor-associated diabetes mellitus occurred in combination with a CTLA-4 medication [11]. Our patient was consistent with these statistics, receiving ipilumumab in addition to nivolumab on days of treatment 1, 21, 42, and 63 as a part of his treatment plan.

Current recommendations per the American Society of Clinical Oncology (ASCO) include measuring glucose levels prior to the initial treatment, and then repeating each treatment cycle for at least six months of therapy [12]. This has proven difficult because patients with symptoms of checkpoint inhibitor-associated diabetes mellitus (CIADM) present abruptly, quickly leading to DKA before detection [8]. Existing literature has documented previous cases of CIADM occurring at different timelines. The median time to onset of endocrine toxicity with PD-1 inhibitors is 14.5 weeks [12].

A review of the current literature showed although that is the median, cases range from a few weeks to over a year before the onset of DKA. One case report detailed DKA after six months of treatment [8], with stable glucose levels below 125 mg/dL for several years prior to initiating treatment. However, our patient did have an HbA1c of 8.1%, suggesting elevated blood sugars and a potential predisposition to CIADM before initiation of nivolumab [8]. Another case report detailed DKA occurring after 14 months of treatment, without documentation of glucose status prior to presentation. Other reports occurred at three months and after just three weeks of therapy [13], suggesting variable onset of CIADM post-initiation of treatment with PD-1 inhibitors.

Our patient had a longer onset of DKA than the median at over 14 months. He was previously diagnosed with adrenal insufficiency secondary to nivolumab, receiving replacement therapy prior to his vascular surgery. There is potential that this fact alone led to a predisposition to developing DKA, but it was not noticed earlier due to his need for stabilization post-surgically. Further, our patient was complicated by his relative immobility post-surgery which made the delirium harder to discern from general fatigue and normal recovery.

New-onset hyperglycemia due to PD-1 inhibitors is difficult to discern from variations in blood sugar due to other causes such as insulin resistance. Current recommendations encourage clinicians to closely investigate patients without risk factors for type 2 diabetes mellitus, such as previous disease or corticosteroid exposure, as fluctuations in their glucose levels should raise clinical suspicion for CIADM [12]. Further, clinicians should have higher clinical suspicion in those with a predisposition to developing diabetes, as their predisposition may lead to earlier development of CIADM [8].

Initial stabilization of the patient after diagnosis included consultation with an endocrinologist, insulin replacement therapy, fluid resuscitation, and electrolyte replacement, as recommended by current guidelines [14]. Patients who develop CIADM often have irreversible destruction of islet cells, requiring treatment with insulin long-term, as was the case in our patient [8].

## Conclusions

This case report highlights a rare adverse event of nivolumab-induced DKA in a patient with a history of autoimmune disease. Prompt recognition and management of this potentially life-threatening complication are crucial. Healthcare providers should be aware of this adverse event and closely monitor patients receiving nivolumab therapy for signs and symptoms of DKA. Shared decision-making regarding the risks and benefits of nivolumab therapy should be undertaken with patients, considering their individual risk factors for developing DKA. Further research is required to assess the side effects of nivolumab and to predict which patients are at risk for adverse events.
